# Effect of opioid-free versus opioid-based anaesthesia among surgical patients in public health care centers: A community medicine perspective

**DOI:** 10.6026/973206300214393

**Published:** 2025-12-15

**Authors:** Anushree Shukla, Naikey Minarey, Mona Bhalavi, Purvi Jain

**Affiliations:** 1 Department of Anesthesiology, Apollo Hospitals, Indore, Madhya Pradesh, India; 2 Department of Pediatrics, Sunderlal Patwa Government Medical College, Mandsaur, Madhya Pradesh, India; 3 Department of Anesthesiology, Government Medical College, Seoni, Madhya Pradesh, India; 4 Department of Anaesthesiology, CARE CHL, Indore, Madhya Pradesh, India

**Keywords:** Opioid-free anaesthesia, multimodal analgesia, community healthcare, postoperative pain, cost-effectiveness

## Abstract

Anxieties about the opioid epidemic should motivate research into more secure and economical methods of managing pain during surgery. Hence, a total of 204 surgical patients receiving either opioid-based anesthesia (OBA) or opioid-free anesthesia (OFA) at public healthcare institutions were compared. Dexmedetomidine, ketamine and lidocane were used in OFA to lessen the severity of postoperative pain, side effects and recovery time. The OFA group also had higher rates of patient satisfaction and overall affordability. Thus, OFA is a more practical and superior substitute for community surgical services.

## Background:

As a whole, the opioid epidemic has caused a fundamental rethinking of how peri-operative pain management is approached, particularly within the framework of public healthcare systems' services, which impact several demographics [1]. For many years, opioid-based anesthesia (OBA) was the standard for managing postoperative pain after surgery. However, concerns about respiratory depression, postoperative nausea and vomiting (PONV), prolonged recalls and dependence prompted researchers to look for alternatives [2, 3]. In situations with limited resources in the public healthcare systems, these complications manifest as long periods of stay in hospitals, higher costs of healthcare and lower-quality care delivery to underserved groups [4]. Opioid-free anaesthesia (OFA) can be viewed as an alternative approach to treatment based on the principle of multimodal analgesia where several non-opioid drugs are used to ensure sufficient pain management at the least possible level of side effects caused by opioids [5]. Various pain pathways are targeted by OFA medications, which may include dexmedetomidine, ketamine, lidocaine and non-steroidal anti-inflammatory medicines (NSAIDs). These treatments have synergistic analgesic effects and can be delivered at different intervals [6, 7]. Recent reports in tertiary care units have reported good results with OFA protocols, such as the decrease in opioid use, pain rating and the occurrence of PONV [8, 9]. When seen through the lens of community medicine, the potential and obstacles for implementing OFA in public healthcare institutions are extraordinary. The poor, who are disproportionately likely to have limited access to postoperative treatment and a higher risk of opioid adverse events, are often seen in community healthcare facilities [10]. In addition, these institutions often face financial constraints, necessitating treatments that are both cost-effective and have shown clinical performance [11]. According to Beloeil and colleagues, OFA significantly reduces morphine use after major abdominal surgery [12], while Mulier found that OFA is feasible for patients having bariatric surgery [13]. Several studies have looked at the effectiveness of OFA in certain surgical groups. Although there is a growing body of research in support of OFA under controlled academic settings, there is a considerable gap in research with reference to its application and effectiveness in the context of the public health care sector as a provider of health care to a diverse community population [14]. The question of how publicly-funded universities with limited resources might benefit from the work of privately-funded postsecondary institutions remains unanswered [15]. Furthermore, there is a lack of research that offers comprehensive analysis from a community medicine viewpoint, including clinical results, patient satisfaction and economic analysis [16]. Therefore, it is of interest to effect of opioid-free versus opioid-based anaesthesia among surgical patients in public health care centers.

## Materials and Methods:

In order to determine the appropriate sample size, we used the method for comparing two independent means. We anticipated a 1.5-point mean difference in VAS pain ratings, a 2.5-point standard deviation and an alpha error of 0.05 and a power of 80%. The result was that each group needed 88 patients. The ultimate sample size was calculated to be 204 individuals, with 102 patients in each group, after accounting for an estimated dropout rate of 15%.

## Participant selection:

## Inclusion criteria:

In order to have elective abdominal or orthopedic procedures under general anesthesia, patients must be adults (18-65 years old), in good health according to the American Society of Anesthesiologists (ASA) standards and able to provide their informed permission. The surgeries typically last between one and four hours.

## Exclusion criteria:

Patients should not be included if they have a history of pain perception disorders, are known to have an allergy to or be incompatible with the study medications, have a history of chronic opioid use or substance abuse, have severe cardiovascular disease (ejection fraction <40%), renal impairment (creatinine clearance <50 mL/min), hepatic dysfunction (Child-Pugh class B or C), have undergone emergency surgeries, are pregnant or lactating, or if they refuse to participate.

## Randomization and Blinding:

The OFA and OBA groups were assigned to eligible patients at random using computer-generated random number sequences with a block size of 10. Using opaque, sealed envelopes that were numbered consecutively allowed us to preserve allocation secrecy. Despite the fact that the anesthesiologist running the procedure was unable to be blinded, postoperative assessors and data analyzers were kept in the dark about group allocation.

## Anaesthesia Protocols:

## Opioid-Free Anaesthesia (OFA) Group:

Two hours before surgery, patients were given the normal premedication of 1000 mg of oral acetaminophen and 200 mg of celecoxib. In order to induce anesthesia, the following were administered: propofol (2-3 mg/kg), a loading dose of dexmedetomidine (1 µg/kg over 10 minutes), ketamine (0.5 mg/kg) and rocuronium (0.6 mg/kg). During maintenance, the patient was given sevoflurane at an end-tidal concentration of 1-2 percent, dexmedetomidine at a rate of 0.4-0.7 µg/kg/h, ketamine at a rate of 0.25 mg/kg/h and lidocaine at a rate of 1.5 mg/kg/h. Supplementing intraoperative analgesia, 0.25% bupivacaine was injected into the surgical site.

## Opioid-Based Anaesthesia (OBA) Group:

To induce anesthesia, patients were given propofol (2-3 mg/kg) and fentanyl (2 µg/kg). Following this, rocuronium (0.6 mg/kg) was administered. Ongoing treatment consisted of sevoflurane at a concentration of 1-2 percent at the end-tidal level and fentanyl boluses at intervals of 0.5-1 mg/kg, according on hemodynamic criteria. Patients were given morphine boluses or patient-controlled analgesia after surgery.

## Primary outcomes:

Quantification of postoperative pain as assessed by the Visual Analogue Scale (VAS, 0-10) at2,6,12 and 24 hours; total analgesic use in morphine equivalents during the first 24 hours; duration until the first request for analgesics was made; and duration of recovery time in the post-anesthesia care unit (PACU).

## Secondary outcomes:

Incidence of PONV, respiratory problems, hemodynamic instability requiring intervention, patient satisfaction score (0-10 scale) at 24 hours, delay to ambulation, duration of hospital stay."

## Data collection:

Before surgery, we took patient demographics and clinical histories. Heart rate, blood pressure, oxygen saturation and intake of anaesthetic agents were recorded at 15-minute intervals throughout the operation. At certain intervals after surgery, study assistants who had been blinded to the patients' groups administered evaluations. Using standardized procedures, adverse occurrences were documented. Medications, PACU time and supplementary therapies for complication management all factored into the final price tag.

## Statistical analysis:

Our data analysis was conducted using SPSS 26.0. The Shapiro-Wilk test was used to validate the normality of all continuous variables. To compare continuous variables with regularly distributed distributions, the mean ± standard deviation was used and the independent samples t-test was employed for these comparisons. We performed frequency and percentage analyses using Fisher's exact tests and chi-square tests, respectively, for categorical data. Any p-value below 0.05 was considered statistically significant. After being randomly chosen, all of the individuals had their "intention-to-treat" analyses performed.

## Results:

In all, 204 patients met the inclusion and randomization criteria, with 102 assigned to each group. After randomization, three patients from the OBA group were removed from further analysis (two of them withdrew permission and one had their operation cancelled), leaving 102 patients from the OFA group and 99 patients from the OBA group for final analysis. From the outset, the groups were quite similar (Table 1 - see PDF). Postoperative pain scores were significantly lower in the OFA group at all measured time points. At 6 hours postoperatively, the OFA group reported mean VAS scores of 3.2 ± 1.4 compared to 4.8 ± 1.7 in the OBA group (p<0.001). This difference persisted at 24 hours (2.1 ± 1.2 vs. 3.6 ± 1.5, p<0.001). Total analgesic consumption in morphine equivalents during the first 24 hours was significantly lower in the OFA group (8.6 ± 4.2 mg vs. 18.4 ± 6.8 mg, p<0.001). Time to first analgesic request was longer in the OFA group (6.8 ± 2.4 hours vs. 3.2 ± 1.6 hours, p<0.001). PACU recovery time was markedly shorter in the OFA group (42.3 ± 12.6 minutes vs. 56.8 ± 15.4 minutes, p<0.001) (Table 2 - see PDF). The incidence of PONV was significantly lower in the OFA group (18.6% vs. 38.2%, p=0.002). Respiratory depression requiring intervention occurred in 2.9% of OFA patients compared to 11.1% of OBA patients (p=0.028). Patient satisfaction scores at 24 hours were higher in the OFA group (8.4 ± 1.2 vs. 7.2 ± 1.6, p<0.001). Time to ambulation was shorter in the OFA group (14.2 ± 4.6 hours vs. 18.8 ± 5.4 hours, p<0.001). Length of hospital stay showed no significant difference between groups (2.8 ± 0.9 vs. 3.1 ± 1.2 days, p=0.052) (Table 3 - see PDF).

## Discussion:

There is enough evidence from this prospective comparative analysis to suggest that opioid-free anesthesia is superior to the conventional opioid-based anaesthesia procedures used in public healthcare facilities. The results show that OFA protocols using multimodal analgesia lead to better pain control, fewer side effects, faster recovery, more patient satisfaction and financial benefits; these findings have significant ramifications for community-based surgical services that serve populations with limited resources. Our OFA group had a much lower mean score for postoperative pain, which is in line with the results of the many studies done on the topic of multimodal analgesic techniques [17]. Of procedures using dexmedetomidine and ketamine provide the same or better analgesia than opioid-based methods in major surgeries, according to research by Puentes Garcia and colleagues [18]. The combination of alpha-2 agonism with dexmedetomidine, NMDA receptor antagonism with ketamine and sodium channel blocking with lidocaine may improve the study of nociceptive pathways. When compared to opioid receptor agonism alone, this combination works better [19, 20]. Consistent with the results of Ziemann-Gimmel and colleagues [21] who shown reduced postoperative opioid need with intraoperative lidocaine infusion, our OFA group had a longer latency to first analgesic request (6.8 hours), suggesting analgesic activity beyond the intraoperative period. From a community medicine standpoint, the significant decline in PONV prevalence is an important and noteworthy outcome. Our rates of 18.6% in the OFA group and 38.2% in the OBA group are similar, which is in agreement with the systematic review that was carried out by Wei Q & Xia M. They found that opioid exposure was the most modifiable risk factor of PONV [22]. Health care costs and patient satisfaction would both be positively affected by a decrease in the usage of antiemetic drugs and an acceleration of the recovery period for patients experiencing postoperative nausea and vomiting (PONV) [38]. An important safety issue, especially in institutions without enough rigorous monitoring systems, will be addressed by the considerable decrease in cases of respiratory depression in our OFA group (2.9% vs. 11.1%) [24]. A major impact on the public healthcare institution can result from the large discrepancy in recovery time between the OFA group and the control group (42.3 vs. 56.8 minutes), given that the PACU capacity is often a stumbling block in the surgical throughput. The issue of high surgical wait times in public healthcare systems might be immediately addressed if the recovery period could be reduced by 25%. This would allow for more procedures to be conducted without the need to invest in additional infrastructure [25, 26]. The shortened ambulation time as a sign of improved recovery is in keeping with the Enhanced Recovery After Surgery (ERAS) guidelines, which stress multimodal opioid-sparing analgesia [27, 28]. State healthcare systems are severely underfunded and the offered cost-efficiency research shows that OFA reduces treatment expenses by 16% (287 vs. 342). Although morphine is the more cost-effective of the two drugs used in the OFA protocol-dexmedetomidine and ketamine-the overall cost-benefit analysis shows that the former is better since it reduces the need to treat problems, keeps patients out of the PACU for longer and uses less rescue medication [29]. Previous health economic analyses of multimodal analgesia have shown cost savings as a result of fewer complications and shorter hospital stays [30].

When comparing the two groups, it is clear that the OFA group received higher-quality treatment, as their patient satisfaction levels were 8.4 vs. 7.2. This finding has important implications for community medicine. When it comes to public healthcare systems serving disadvantaged populations, patient-centered outcomes and clinical objectives should take center stage [31]. This results in better pain management, faster functional recovery and a treatment experience that is more in line with patient values and expectations without the adverse effects of opioids, such as pruritus and urine retention [32]. Our research raises a variety of questions about how to put our results into practice. Only with well-trained personnel, established procedures and quality monitoring systems can OFA protocols be successfully implemented in community healthcare institutions [33]. The medicines employed in our OFA regimen are cost-effective, do not need special storage conditions and may be safely delivered by a qualified anesthetist, making them suitable for usage in a range of settings [34]. However, it is also important to carefully choose individuals, since some patient populations (such as the elderly or those with cardiovascular disease) may need protocol adjustments [35]. Some limitations exist in our investigation. The selected 24-hour follow-up period neglected to consider the potential long-term effects, such as the onset of persistent post-surgical pain, which may be significantly worsened by opioid exposure [36]. Because the study only included controlled elective procedures, the findings may not be generalizable to more complicated or urgent surgical procedures. Furthermore, not only were the assessors blindfolded, but the treatments themselves did not permit the complete blinding procedure. Because this study only included patients with ASA levels of I-II, the results may not apply to those at higher risk. Finally, we have only included direct medical expenditures in our cost analysis; we have not accounted for societal costs, such as productivity losses or long-term consequences connected to opioids. Future study should include larger surgery groups, evaluate the long-term effects including opioid usage and chronic pain and design thorough health economics studies using quality-adjusted life years. It is possible that the optimal methods for scaling OFA protocols in different public healthcare contexts can be uncovered by implementation science research [37]. Additionally, personalized anesthesia techniques might be achieved by study on patient-specific characteristics that predict the optimal response to OFA instead of OBA [38].

## Conclusion:

A safe and cost-effective alternative to the traditional opioid-based methods of anesthesia for surgical patients at publicly-funded hospitals is opioid-free anesthesia (OFA). Because of its multimodal design, it lessens the likelihood of side effects while also ensuring better pain management, faster recovery and patient satisfaction. Community health care systems' surgical treatment, both in terms of quality and efficiency, might benefit greatly from the adoption of standardized OFA techniques.
